# Comparison between conduction system pacing and cardiac resynchronization therapy in right bundle branch block patients

**DOI:** 10.3389/fphys.2022.1011566

**Published:** 2022-09-21

**Authors:** Marina Strocchi, Karli Gillette, Aurel Neic, Mark K. Elliott, Nadeev Wijesuriya, Vishal Mehta, Edward J. Vigmond, Gernot Plank, Christopher A. Rinaldi, Steven A. Niederer

**Affiliations:** ^1^ School of Biomedical Engineering and Imaging Sciences, King’s College London, London, United Kingdom; ^2^ BioTechMed-Graz, Graz, Austria; ^3^ Gottfried Schatz Research Center, Medical University of Graz, Graz, Austria; ^4^ NumeriCor GmbH, Graz, Austria; ^5^ Guy’s and St Thomas’ NHS Foundation Trust, London, United Kingdom; ^6^ Lyric, University of Bordeaux, Bordeaux, France

**Keywords:** heart failure, dyssynchrony, conduction system pacing, cardiac resynchronization therapy, right bundle branch block, his bundle pacing, left bundle pacing

## Abstract

A significant number of right bundle branch block (RBBB) patients receive cardiac resynchronization therapy (CRT), despite lack of evidence for benefit in this patient group. His bundle (HBP) and left bundle pacing (LBP) are novel CRT delivery methods, but their effect on RBBB remains understudied. We aim to compare pacing-induced electrical synchrony during conventional CRT, HBP, and LBP in RBBB patients with different conduction disturbances, and to investigate whether alternative ways of delivering LBP improve response to pacing. We simulated ventricular activation on twenty-four four-chamber heart geometries each including a His-Purkinje system with proximal right bundle branch block (RBBB). We simulated RBBB combined with left anterior and posterior fascicular blocks (LAFB and LPFB). Additionally, RBBB was simulated in the presence of slow conduction velocity (CV) in the myocardium, left ventricular (LV) or right ventricular (RV) His-Purkinje system, and whole His-Purkinje system. Electrical synchrony was measured by the shortest interval to activate 90% of the ventricles (BIVAT-90). Compared to baseline, HBP significantly improved activation times for RBBB alone (BIVAT-90: 66.9 ± 5.5 ms vs. 42.6 ± 3.8 ms, *p* < 0.01), with LAFB (69.5 ± 5.0 ms vs. 58.1 ± 6.2 ms, *p* < 0.01), with LPFB (81.8 ± 6.6 ms vs. 62.9 ± 6.2 ms, *p* < 0.01), with slow myocardial CV (119.4 ± 11.4 ms vs. 97.2 ± 10.0 ms, *p* < 0.01) or slow CV in the whole His-Purkinje system (102.3 ± 7.0 ms vs. 75.5 ± 5.2 ms, *p* < 0.01). LBP was only effective in RBBB cases if combined with anodal capture of the RV septum myocardium (BIVAT-90: 66.9 ± 5.5 ms vs. 48.2 ± 5.2 ms, *p* < 0.01). CRT significantly reduced activation times in RBBB in the presence of severely slow RV His-Purkinje CV (95.1 ± 7.9 ms vs. 84.3 ± 9.3 ms, *p* < 0.01) and LPFB (81.8 ± 6.6 ms vs. CRT: 72.9 ± 8.6 ms, *p* < 0.01). Both CRT and HBP were ineffective with severely slow CV in the LV His-Purkinje system. HBP is effective in RBBB patients with otherwise healthy myocardium and Purkinje system, while CRT and LBP are ineffective. Response to LBP improves when LBP is combined with RV septum anodal capture. CRT is better than HBP only in patients with severely slow CV in the RV His-Purkinje system, while CV slowing of the whole His-Purkinje system and the myocardium favor HBP over CRT.

## 1 Introduction

Cardiac resynchronization therapy (CRT) is an effective treatment for heart failure (HF) patients with dyssynchrony. (Sieniewicz et al., 2019). Treatment is delivered with a right ventricular (RV) lead and a left ventricular (LV) lead implanted in a tributary of the coronary sinus (CS), normally targeting the latest activated region. Clinical trials consistently show that CRT benefits patients with left bundle branch block (LBBB). On the other hand, observational studies reported higher mortality rates in RBBB vs. LBBB patients following CRT (Auricchio et al., 2014). Regardless, between 9% and 13% of patients recruited in major CRT clinical trials have right bundle branch block (RBBB) (Salden O. A. E. et al., 2020).

Despite the lack of evidence of positive outcome in RBBB patients following CRT, (Henin et al., 2020) recent observational retrospective studies reported potential benefits in subgroups of the RBBB population.(Pastore et al., 2018). As CRT aims to correct LV activation delay, patients with concomitant LV activation delay and RBBB, manifesting as atypical RBBB, might still benefit from CRT. Pastore et al. (2018) reported that atypical RBBB led to response to CRT in 71.4% of cases, opposed to only 19.4% of cases in the presence of typical RBBB. Patients with RBBB concomitant with left anterior fascicular block (LAFB) or left posterior fascicular block (LPFB) might also potentially benefit from CRT, as an anterior or posterior block induces partially delayed LV activation (Naruse et al., 2014; Dennis et al., 2022). Identifying specific RBBB patient subgroups that will benefit from CRT remains an ongoing clinical challenge.

Conduction system pacing (CSP), delivered through His bundle (HBP) or left bundle pacing (LBP), has emerged as a valuable alternative treatment to standard CRT for LBBB patients (Lustgarten et al., 2015; Arnold et al., 2018). HBP has recently been applied to RBBB patients with impaired LV function with 95% success rates, with QRS narrowing achieved in 78% of patients (Sharma et al., 2018). However, HBP has the disadvantages of being technically challenging and requiring high pacing thresholds. LBP overcomes these issues, but it is often associated with RV delayed activation (Strocchi et al., 2020b). In RBBB patients with CRT indication, Vijayaraman et al. (2022) found that LBP led to moderate QRS narrowing, less so than with HBP. Nevertheless, RBBB correction and attenuation was observed in 33% and 64% of patients, respectively. The authors provided two hypotheses through which LBP reduced RV delayed activation: 1) non-selective capture of the LBP stimulus or 2) anodal capture of the ring electrode, when this is in good contact with the RV side of the septum. Although several studies showed that CSP is an efficient CRT delivery method for LBBB patients, additional data is needed to understand the effect of CSP on RBBB patients.

We aim to study the effects of CRT, HBP, and LBP on different RBBB patient groups using computational models. Ventricular activation was simulated on twenty-four heart geometries generated from HF patients with CRT indication. We performed simulations in the presence of proximal RBBB combined with the following conduction disturbances: LAFB, LPFB, slow LV His-Purkinje system conduction velocity (CV), slow RV His-Purkinje system CV, slow His-Purkinje system CV in both ventricles and slow myocardium CV. We additionally tested the effect of non-selective LBP and selective LBP combined with anodal capture of the RV septum to test which approach results in correction or attenuation of the RBBB activation pattern.

## 2 Materials and methods

We used twenty-four four-chamber heart tetrahedral meshes generated from HF patients as part of a previous study, with a mesh resolution of 1 mm (Strocchi et al., 2020a). Table 1 presents the patient cohort demographics, the LV, RV volume, and LV diameter derived from the mesh as described in (Strocchi et al., 2020a). A His-Purkinje network accounting for three LV fascicles (anterior and posterior, which further branches into two septal fascicles) and two RV fascicles (one septal fascicle and the moderator band) was generated for each mesh as described previously (Gillette et al., 2021; Gillette et al., 2022). In the Supplement, we provide additional details about the construction of the His-Purkinje networks. Proximal RBBB was introduced by disconnecting the right bundle from the RV Purkinje system along the His. LAFB and LPFB were introduced by disconnecting the anterior and the posterior fascicle from the left bundle, respectively.

**TABLE 1:** Patient Characteristics. The table shows the characteristics of the twenty-four heart failure patients the meshes were derived from. The LV volume, the RV volume and the LV diameter were derived from the meshes. All quantities apart from the sex are presented as mean ± standard deviation.

patient characteristics	
Age [years]	67 ± 14
Sex	23 Males, 1 Female
LV volume [ml]	269 ± 78
RV volume [ml]	219 ± 39
LV diameter [mm]	65 ± 8

### 2.1 Electrophysiology simulations

An Eikonal model was used to simulate ventricular electrical activation (Neic et al., 2017). The Eikonal model computes the local time t_a_(**x**) at each node with location **x** within a domain Ω, provided an initial activation time t_0_ at an initial stimulus location Γ and the CV tensor **V**, containing the squared CV along the fiber, sheet and normal to sheet directions.

$$\sqrt{\nabla t_{a}{\left(x\right)}^{T}V\nabla t_{a}\left(x\right)}=1,x\in \Omega$$

$$t_{a}\left(x\right)=t_{0},x\in \Gamma$$

The myocardium was treated as a transversely isotropic medium, so the CV in the sheet and in the normal directions were set to be the same and are referred to as transverse CV. The CV of ventricular myocardium was set to 0.6 m/s and 0.24 m/s in the fiber and transverse directions, respectively. (Draper and Mya-Tu, 1959). The His-Purkinje system was assigned with a CV of 3.0 m/s (Ono et al., 2009). During sinus rhythm, the CV of each fascicle was tuned to achieve simultaneous activation of the end of the three LV fascicles, and activation of the end of the two RV fascicles 10 ms later than the LV fascicles, (Gillette et al., 2022) to replicate the Durrer maps (Durrer et al., 1970). The Eikonal equation was solved with the Fast Iterative Algorithm, as described in (Neic et al., 2017).

**FIGURE 1:**
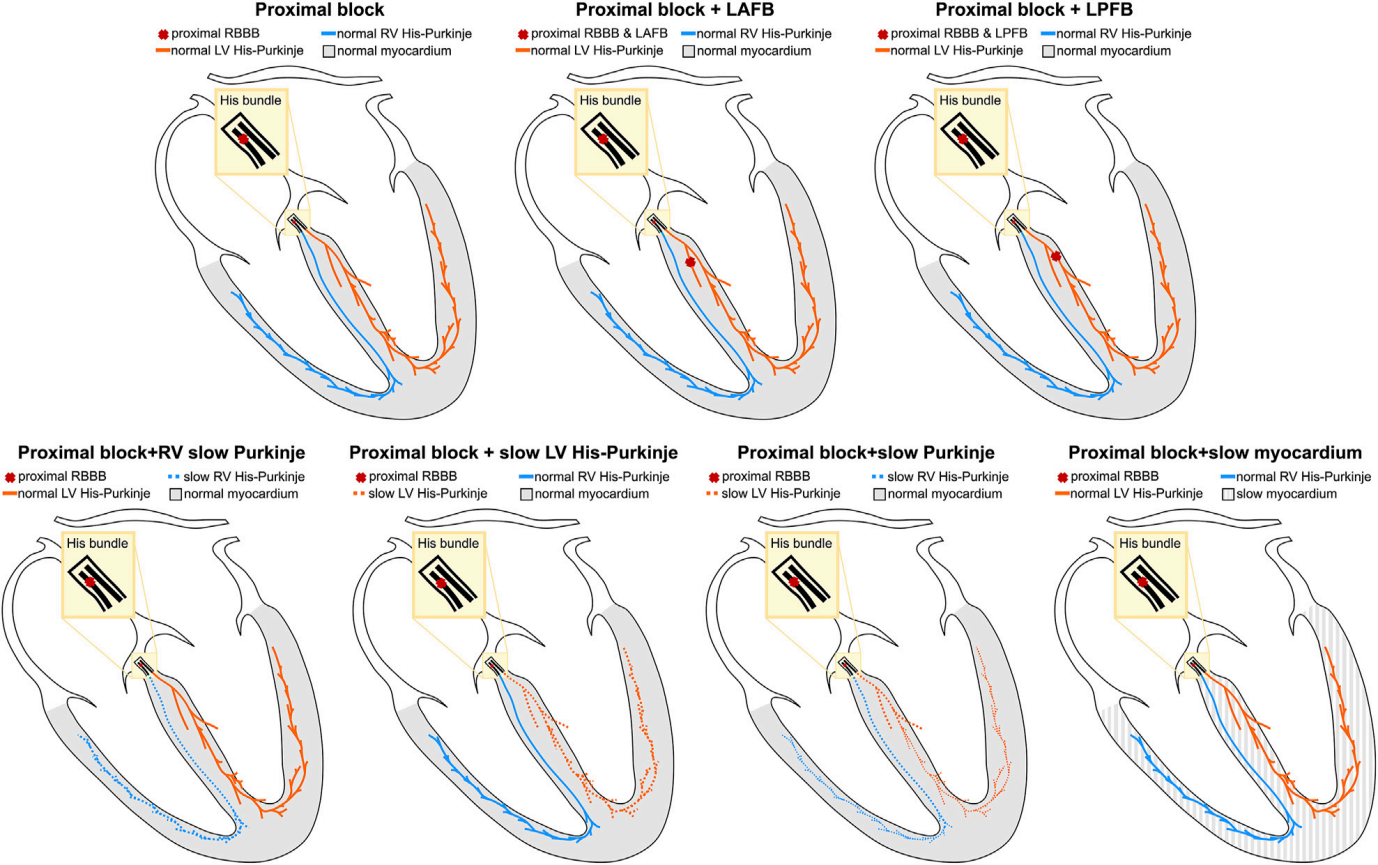
Schematic representation of conduction disturbances. Conduction blocks in the proximal RBBB or in the left anterior or posterior fascicles are represented by red crosses. The myocardium, the left and the right His-Purkinje system are shown in grey, orange and blue, respectively.

RBBB patients may present with additional conduction disturbances such as LAFB, LPFB (Naruse et al., 2014; Dennis et al., 2022), and His-Purkinje (Maguy et al., 2009) or myocardium CV slowing (Akar et al., 2004), which were both reported in the failing heart due to altered gap junction protein expression. To represent the heterogeneity of the RBBB population, we simulated the following scenarios: 1) proximal RBBB and otherwise normal myocardium and His-Purkinje system; 2) proximal RBBB and LAFB; 3) proximal RBBB and LPFB; 4) proximal RBBB with mild or severe LV His-Purkinje CV slowing; 5) proximal RBBB with mild or severe RV His-Purkinje CV slowing; 6) proximal RBBB with mild or severe His-Purkinje CV slowing; 7) proximal RBBB with mild or severe myocardium CV slowing. Mild and severe CV slowing were simulated by reducing the CV down to 70% and 35% of reference CV values. Figure 1 summarizes the different conduction disturbances we considered in this study. In the Supplement, we show the simulated activation times at baseline for proximal RBBB, and proximal RBBB concomitant with LAFB or LPFB for all twenty-four heart geometries. We then compare the simulated activation patterns and activation metrics against reported RBBB activation features and values in the literature, to show that the models are able to replicate RBBB activation patterns.

**FIGURE 2:**
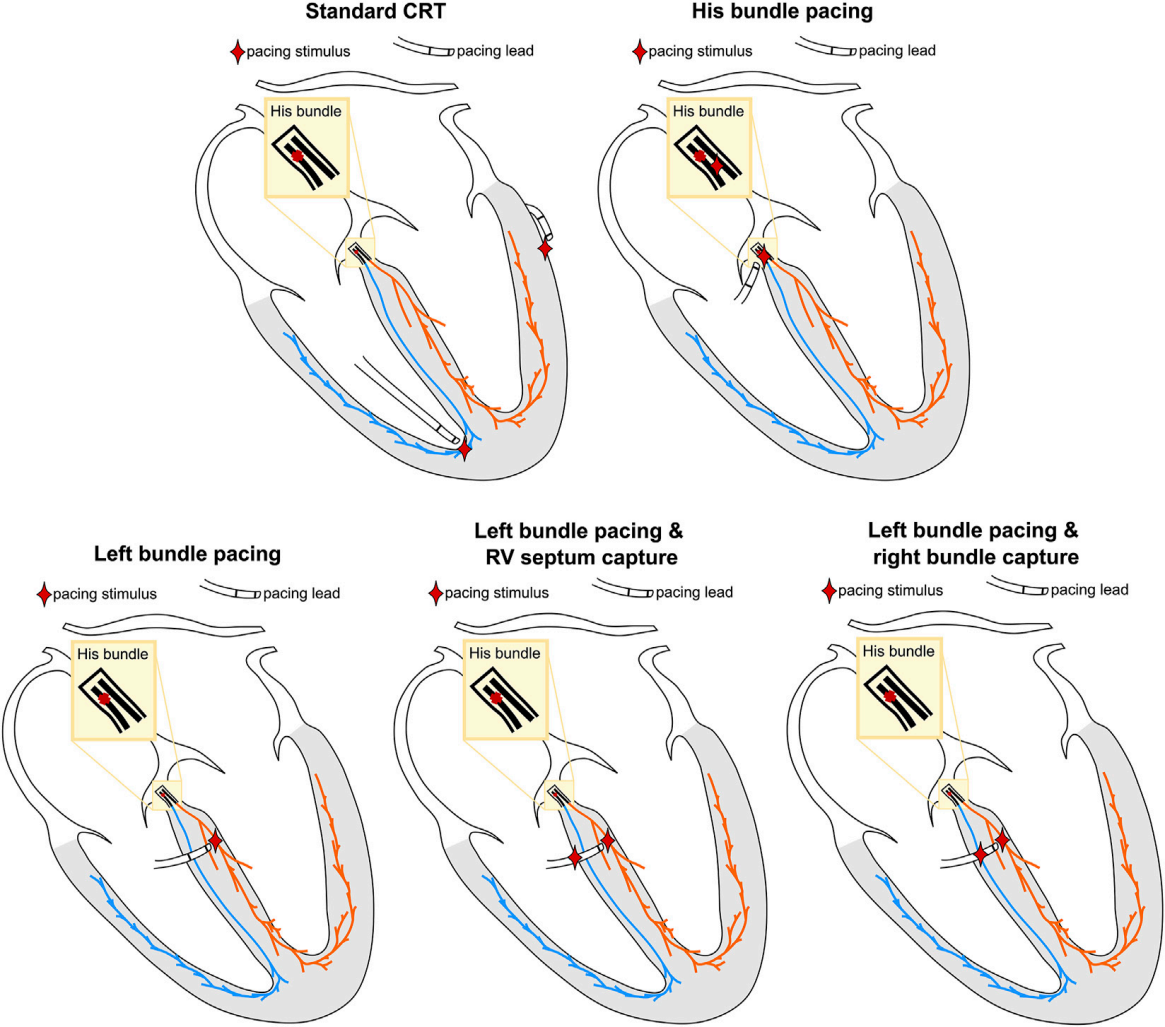
Schematic representation of the simulated pacing locations. The location of the pacing stimuli is represented by the red star. The colors of the different regions correspond to Figure 1.

Selective HBP was simulated by pacing the His below the block, to simulate perfect correction of proximal RBBB. Selective LBP was simulated by stimulating the left bundle. For non-selective LBP, the LBP stimulus was extended to the surrounding myocardium. Anodal capture of the RV septum during LBP was simulated by projecting the LBP stimulus site onto the RV septum. This site and the left bundle were then paced simultaneously. To investigate the difference between anodal capture of RV septum myocardium and distal right bundle anodal capture, we moved the RV septum stimulus to the closest point on the right bundle and paced simultaneously to selective and non-selective LBP. Finally, standard CRT was simulated by stimulating the RV at the apex and the LV at the latest activated region in the LV epicardium, with the RV-LV delay set to 0 ms. Figure 2 shows a schematic representation of the pacing locations.

We quantified biventricular (BIV) synchrony using the 90% of biventricular activation time (BIVAT-90), computed as the shortest interval needed to activate 90% of the ventricles, and the biventricular dyssynchronous index (BIVDI), computed as the standard deviation of ventricular activation times. The BIVDI was computed accounting for the activation times of all nodes of the ventricles to quantify the dispersion of ventricular activation, based on clinical studies using activation time measurements from electrocardiographic imaging (Arnold et al., 2018) or an ECG belt (Salden F. C. W. M. et al., 2020). LV and RV synchrony were assessed by computing 95% of LV activation time (LVAT-95) and the LV dyssynchronous index (LVDI), and the 95% of RV activation time (RVAT-95) and the RV dyssynchronous index (RVDI), respectively. The peri-annular regions of the interventricular valves were excluded when computing activation times, while the septum was considered to be part of the LV when computing LV metrics.

### 2.2 Statistical analysis

Simulation results were compared using one-way analysis of variance (ANOVA). Post-hoc comparison analysis was performed to see which pairwise comparisons were statistically different using the Tukey’s honestly significant difference test.

## 3 Results

**FIGURE 3:**
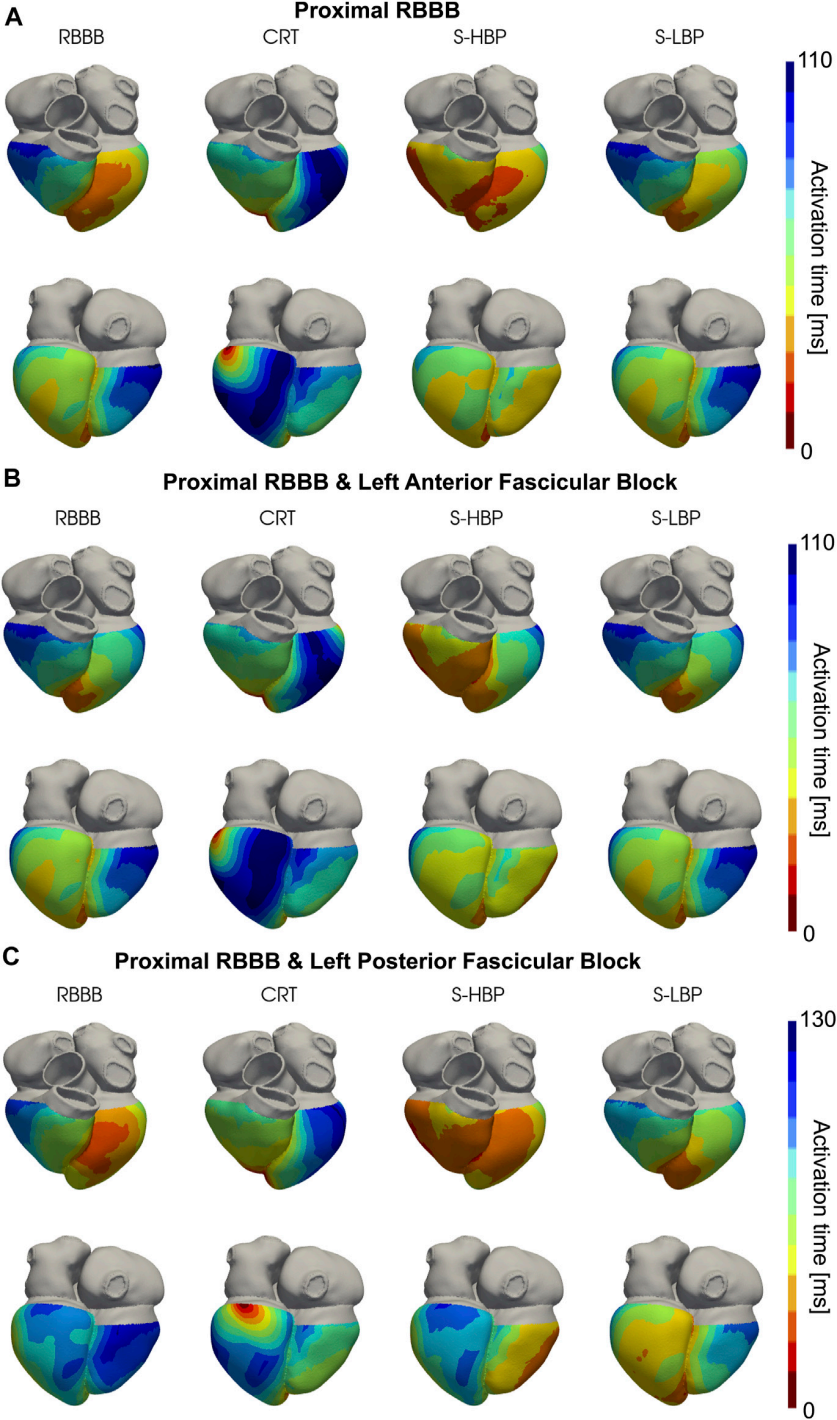
Simulated Activation Times: Activation times simulated on one of the patients for baseline, CRT, selective HBP (S-HBP) and selective LBP (S-LBP) in the presence of proximal RBBB combined with: **(A)** otherwise normal His-Purkinje system, **(B)** left anterior fascicular block and **(C)** left posterior fascicular block.

**FIGURE 4:**
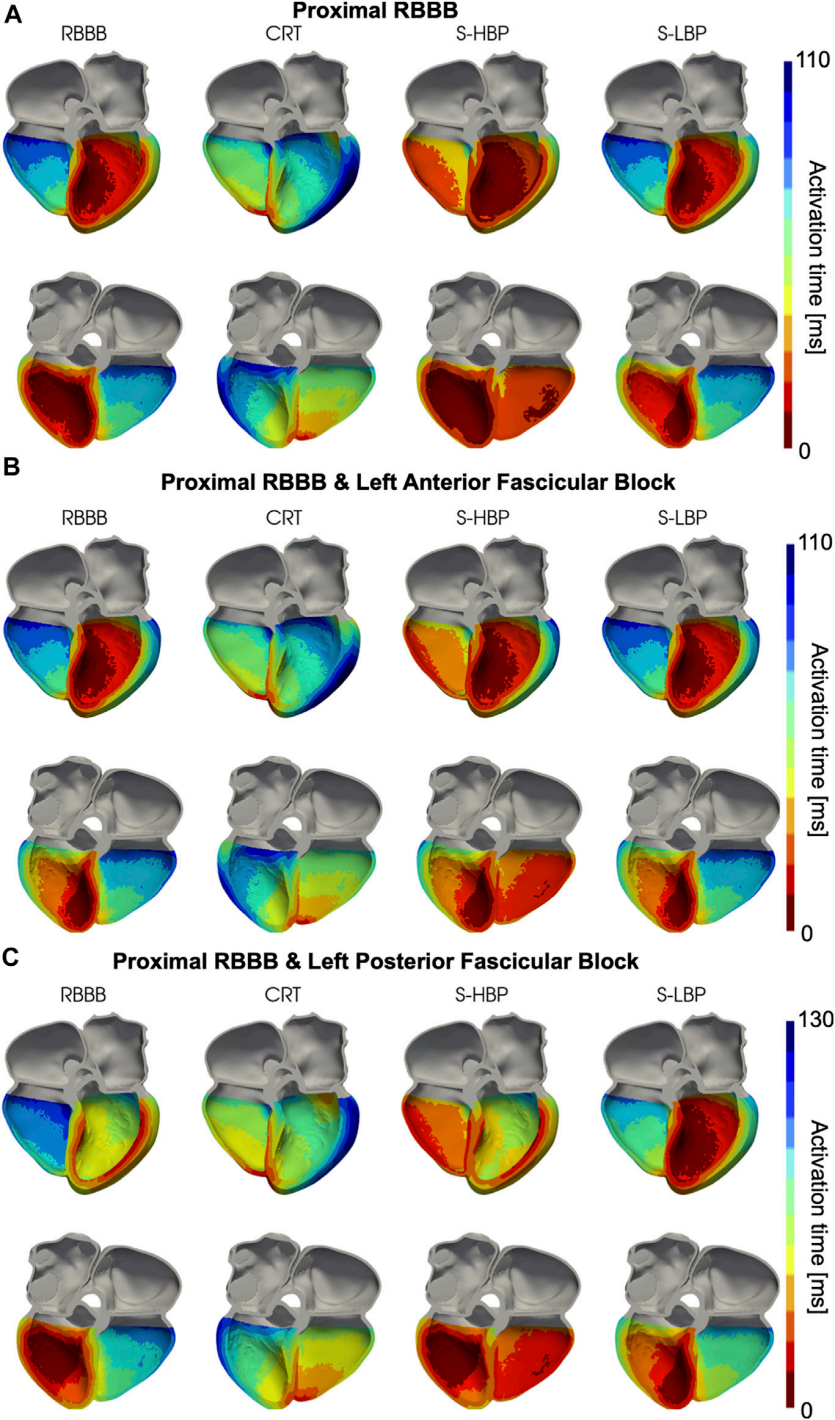
Simulated Activation Times (clipped view): Activation times simulated on one of the patients for baseline, CRT, selective HBP (S-HBP) and selective LBP (S-LBP) in the presence of proximal RBBB, shown on an anterior and a posterior clipped view of the mesh to show activation times. Proximal RBBB was combined with: **(A)** otherwise normal His-Purkinje system, **(B)** left anterior fascicular block and **(C)** left posterior fascicular block.

We simulated proximal RBBB with otherwise normal His-Purkinje system, and combined with LAFB or LPFB. Figures 3, 4 show the distribution of simulated activation times for these three different scenarios during baseline, standard CRT, selective HBP and selective LBP for one heart geometry, with red and blue areas representing early and late activated regions, respectively. RV delayed activation caused by RBBB at baseline with otherwise healthy His-Purkinje conduction (Figure 3A), LAFB (Figure 3B) or LPFB (Figure 3C) was corrected by HBP. Septal activation, which during baseline occurs from left to right due to RBBB, is synchronized by HBP as the electrical propagation can travel along the right bundle, activating the RV septal fascicle and the RV moderator band (Figure 4, columns 1 and 3). On the other hand, CRT led to prolonged LV activation times in all three cases. Selective LBP preserved baseline RV delayed activation in the presence of proximal RBBB alone and RBBB combined with LAFB, while it improved activation when RBBB was concomitant with LPFB, because the LBP stimulus was placed downstream from the LPFB. Figure 4C shows that, at baseline, the posterior septum and the posterior LV free wall are activated late due to LPFB. As opposed to HBP, where late activation of the LV posterior septum is preserved, selective LBP is able to activate these areas early, leading to improved LV and RV activation.

**FIGURE 5:**
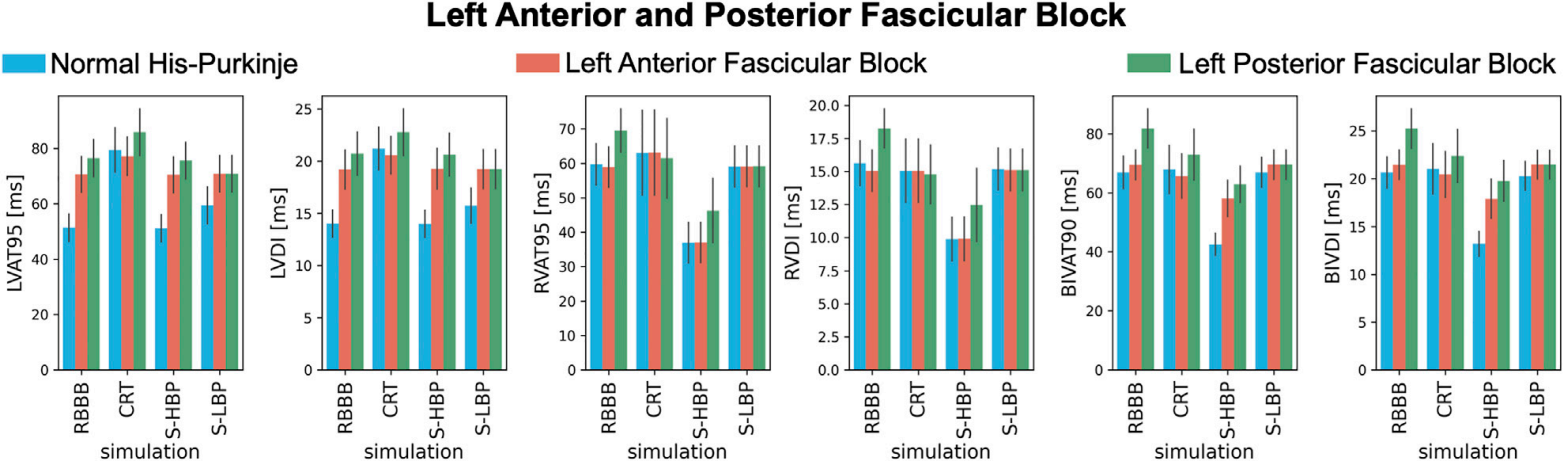
Response with RBBB, left anterior or posterior fascicular block: LVAT-95, LVDI, RVAT-95, RVDI, BIVAT-90, and BIVDI simulated in the presence of proximal RBBB combined with otherwise normal His-Purkinje system (blue bars), left anterior fascicular block (orange bars) and left posterior fascicular block (green bars). Activation was simulated during baseline, standard CRT, selective HBP (S-HBP) and selective LBP (S-LBP). Results are presented as mean ± standard deviation.

These results are confirmed when comparing LV, RV, and BIV response metrics between baseline and pacing (Figure 5). During proximal RBBB alone (Figure 5, blue bars), selective HBP caused no change in LVAT-95 compared to baseline (51.3 ± 4.9 ms vs. 51.2 ± 4.9 ms, *p* = 0.9), while LVAT-95 got worse with CRT (79.4 ± 8.0 ms, *p* < 0.01). On the other hand, RVAT-95 remained unaltered by CRT (59.7 ± 6.0 ms vs. 63.1 ± 12.3 ms, *p* = 0.76) and improved with HBP (36.9 ± 5.8 ms, *p* < 0.01). BIVAT-90 was unaltered by CRT (66.9 ± 5.5 ms vs. 67.9 ± 8.2 ms, *p* = 0.9) and by selective LBP (66.9 ± 5.1 ms, *p* = 0.9) due to prolonged RV activation (RVAT-95: 59.1 ± 6.0 ms, *p* = 0.9 vs. baseline), while HBP shortened BIVAT-90 significantly (42.6 ± 3.8 ms, *p* < 0.01 vs. baseline). When RBBB was concomitant with LAFB (Figure 5, orange bars) or LPFB (Figure 5, green bars), LVAT-95 was worsened by CRT (RBBB + LAFB: 70.6 ± 6.4 ms vs. 77.2 ± 6.9 ms, *p* < 0.01; RBBB + LPFB: 76.5 ± 6.7 ms vs. 85.8 ± 8.5 ms, *p* < 0.01) but remained unchanged during HBP (RBBB + LAFB: 70.4 ± 6.5 ms *P* = 0.9 vs. baseline; RBBB + LPFB: 75.6 ± 6.6 ms *P* = 0.9 vs. baseline). Similar to proximal RBBB alone, in the presence of LAFB RVAT-95 was unaltered by CRT and selective LBP, and significantly improved by selective HBP. Selective LBP improved LVAT-95 (70.8 ± 6.5 ms *P* = 0.04 vs. baseline) and RVAT-95 (59.2 ± 5.9 ms, *p* < 0.01 vs. baseline) with RBBB and LPFB, although differences in LVAT-95 were not statistically significant. In this case, BIVAT-90 was improved significantly by all pacing modalities (baseline: 81.8 ± 6.6 ms vs. CRT: 72.9 ± 8.6 ms *P* < 0.01, HBP: 62.9 ± 6.2 ms *P* < 0.01, LBP: 69.5 ± 5.0 ms *P* < 0.01), but most significantly by HBP. HBP decreased BIVAT-90 with RBBB combined with LAFB as well (69.5 ± 5.0 ms vs. 58.1 ± 6.2 ms, *p* < 0.01), thanks to complete RBBB correction.

**FIGURE 6:**
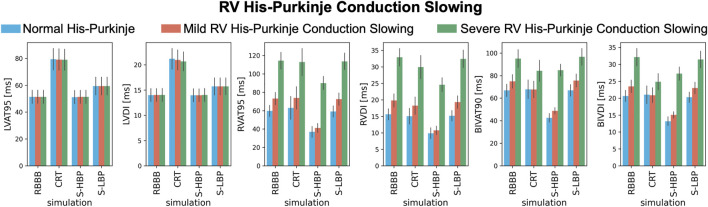
Response with RV His-Purkinje Conduction Slowing: LVAT-95, LVDI, RVAT-95, RVDI, BIVAT-90, and BIVDI simulated in the presence of proximal RBBB combined with otherwise normal His-Purkinje system (blue bars), mild (orange bars) and severe (green bars) RV His-Purkinje conduction slowing. Activation was simulated during baseline, standard CRT, selective HBP (S-HBP) and selective LBP (S-LBP). Results are presented as mean ± standard deviation.

We investigated how mild and severe deterioration of RV His-Purkinje conduction properties affected response to pacing by reducing the RV His-Purkinje CV. Mild RV His-Purkinje conduction slowing combined with proximal RBBB (Figure 6, orange bars) favored HBP (74.9 ± 6.0 ms vs. 48.8 ± 2.8 ms, *p* < 0.01) over CRT, although CRT still shortened BIVAT-90 (67.7 ± 7.4 ms, *p* < 0.01 vs. baseline). In the presence of severe RV His-Purkinje conduction slowing, CRT was effective at shortening BIVAT-90 (95.1 ± 7.9 vs. 84.3 ± 9.3 ms, *p* < 0.01) and RVDI (33.0 ± 2.7 vs. 30.0 ± 3.5 ms, *p* < 0.01), and was comparable to HBP (BIVAT-90: 84.8 ± 5.2 ms, *p* = 0.9 vs. CRT). On the other hand, BIVDI was better with CRT than HBP (baseline: 32.1 ± 2.6 ms vs. CRT: 24.9 ± 2.4 ms vs. 27.3 ± 1.9 ms). LBP was ineffective, as RV activation remained delayed in the presence of both mild (BIVAT90: 75.7 ± 5.7 ms, *p* = 0.9 vs baseline) and severe (96.6 ± 7.5 ms, *p* = 0.9 vs. baseline) RV His-Purkinje conduction slowing.

**FIGURE 7:**
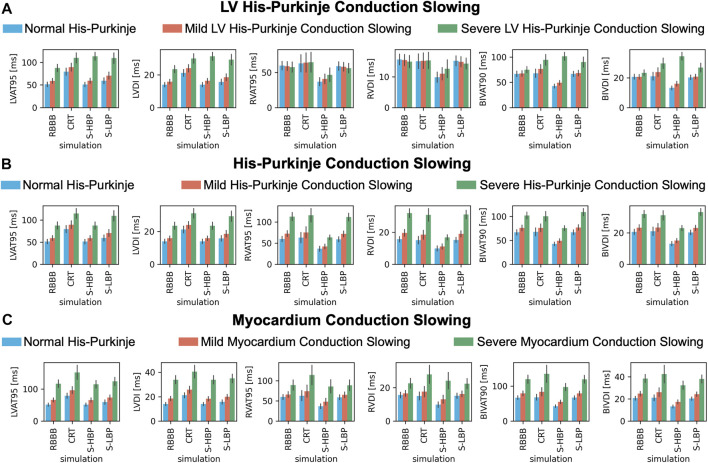
Response with LV His-Purkinje, whole His-Purkinje and myocardium conduction slowing: LVAT-95, LVDI, RVAT-95, RVDI, BIVAT-90, and BIVDI simulated in the presence of proximal RBBB combined with otherwise normal His-Purkinje system (blue bars) and: **(A)** mild (orange bars) and severe (green bars) LV His-Purkinje conduction slowing; **(B)** mild (orange bars) and severe (green bars) His-Purkinje conduction slowing; **(C)** mild (orange bars) and severe (green bars) myocardium conduction slowing. Activation was simulated during baseline, standard CRT, selective HBP (S-HBP) and selective LBP (S-LBP). Results are presented as mean ± standard deviation.

To test how alterations of conduction properties of the LV His-Purkinje system, the whole His-Purkinje system and the myocardium affected response to pacing, we simulated RBBB combined with LV His-Purkinje system CV slowing, His-Purkinje system CV slowing and myocardium CV slowing (Figure 7). Patients with proximal RBBB and mild LV His-Purkinje conduction slowing (Figure 7A, orange bars) responded to HBP. BIVAT-90 and RVAT-95 were significantly better with HBP compared to baseline (BIVAT-90: 67.5 ± 5.5 ms vs. 49.1 ± 4.6 ms, *p* < 0.01; RVAT-95: 59.2 ± 6.3 ms vs. 40.9 ± 6.8 ms, *p* < 0.01), while LVAT-95 remained unaltered (59.1 ± 5.6 ms vs. 59.4 ± 5.6 ms, *p* = 0.9). CRT worsened both LVAT-95 (89.4 ± 8.7 ms, *p* < 0.01) and BIVAT-90 (76.4 ± 8.6 ms, *p* < 0.01) compared to baseline. CRT also led to worse LV, RV, and BIV activation in the presence of severe LV His-Purkinje conduction slowing (Figure 7, green bars). In this case, HBP was also ineffective, leading to longer LVAT-95 (87.6 ± 7.9 ms vs. 113.4 ± 8.7 ms, *p* < 0.01) and BIVAT-90 (75.2 ± 6.1 ms vs. 94.5 ± 10.5 ms, *p* < 0.01) compared to baseline, despite RV activation times were still shortened thanks to RBBB correction (RVAT-95: 57.8 ± 7.1 ms vs. 46.8 ± 9.7 ms, *p* < 0.01). When proximal RBBB was concomitant with mild (Figure 7B, orange bars) or severe (Figure 7B, green bars) conduction slowing of the whole His-Purkinje system, CRT worsened LVAT-95 from baseline, while BIVAT-90 and RVAT-95 remained unchanged. On the other hand, HBP remained effective at shortening BIVAT-90 (severe His-Purkinje conduction slowing: 102.3 ± 7.0 ms vs. 75.5 ± 5.2 ms, *p* < 0.01) and RVAT-95 (112.8 ± 9.5 ms vs. 63.6 ± 5.4 ms, *p* < 0.01). Similarly, HBP shortened BIV and RV activation in the presence of proximal RBBB, normal His-Purkinje conduction but mildly (BIVAT-90: 79.2 ± 6.8 ms vs. 54.8 ± 5.2, *p* < 0.01; RVAT-95: 65.8 ± 7.3 ms vs. 48.2 ± 8.3 ms, *p* < 0.01) or severely (BIVAT-90: 119.4 ± 11.4 ms vs. 97.2 ± 10.0 ms, *p* < 0.01; RVAT-95: 89.4 ± 12.7 ms vs. 86.1 ± 16.3 ms *P* = 0.9) slow myocardium (Figure 7C), although differences in RVAT-95 with severely slow myocardium were not statistically significant. On the other hand, in these cases, CRT worsened LVAT-95, RVAT-95, and BIVAT-90. In particular, BIVAT-90 was significantly increased during CRT compared to baseline in the presence of severe myocardium conduction slowing (CRT: 134.8 ± 24.3 ms, *p* < 0.01 baseline). CRT and LBP were ineffective in the presence of RBBB combined with mild or severe CV slowing affecting the LV His-Purkinje, the whole His-Purkinje or the myocardium. On the other hand, HBP was ineffective only when RBBB was concomitant with severe LV His-Purkinje CV slowing due to delayed LV activation, while it improved LV, RV, and BIV activation times in all other simulated scenarios.

**FIGURE 8:**
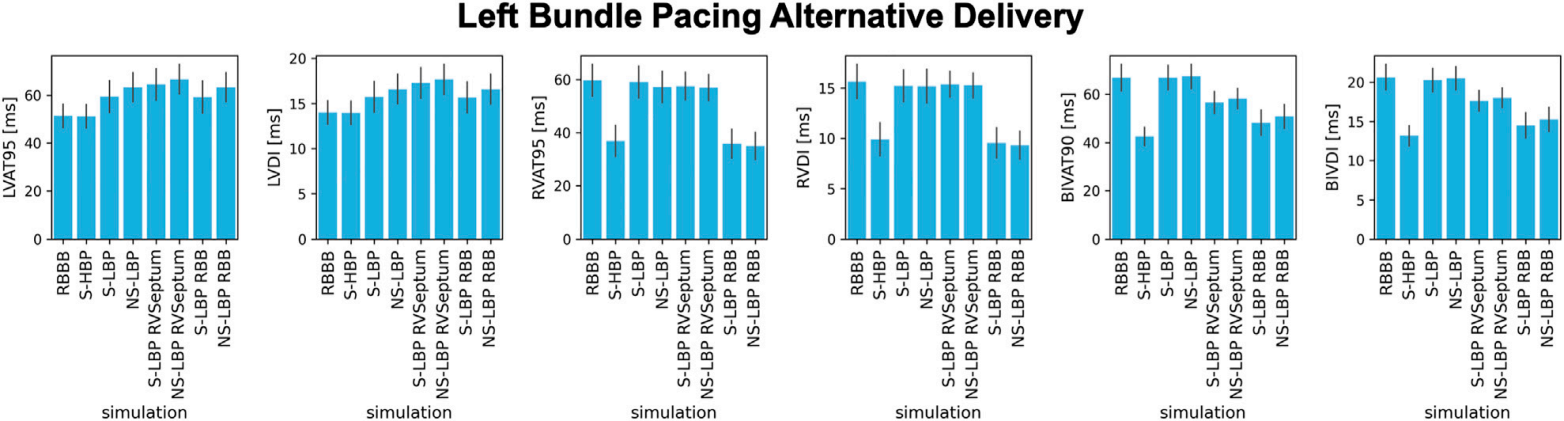
Response to different HBP and LBP delivery methods: LVAT-95, LVDI, RVAT-95, RVDI, BIVAT-90, and BIVDI simulated in the presence of proximal RBBB. Activation was simulated during baseline, selective HBP (S-HBP), selective LBP (S-LBP), non-selective LBP (NS-LBP), selective and non-selective LBP with RV septum myocardial anodal capture (S-LBP RVSeptum and NS-LBP RVSeptum) and selective and non-selective LBP with right bundle anodal capture (S-LBP RBB and NS-LBP). Results are presented as mean ± standard deviation.

In all cases apart from proximal RBBB combined with LPFB, selective LBP did not improve BIV activation times due to prolonged RV activation during pacing. We investigated whether non-selective capture of the left bundle, selective LBP and non-selective LBP combined with anodal capture of the RV septum or the right bundle improved RV activation. Figure 8 shows the results for proximal RBBB baseline, HBP and different delivery methods for LBP. Non-selective LBP was comparable to selective LBP in terms of all metrics (*p* > 0.05). It is however worth noting that, while not statistically significant, non-selective LBP prolongs LVAT-95 compared to selective LBP. This is because, with non-selective pacing, early activation starts in the septum travelling through the slow myocardium, rather than the Purkinje system alone, as opposed to selective LBP. On the other hand, BIVAT-90 were significantly shorter with selective LBP and anodal capture of the RV septum compared to baseline and selective and non-selective LBP alone (baseline: 66.9 ± 5.5 ms, S-LBP: 66.9 ± 5.1 ms vs. NS-LBP: 67.4 ± 5.0 ms vs. S-LBP + RV septum: 56.6 ± 4.5 ms, *p* < 0.01). Biventricular synchrony was also improved with RV septum anodal capture compared to selective and non-selective LBP alone (BIVDI: S-LBP: 20.3 ± 1.5 ms and NS-LBP: 20.5 ± 1.5 ms vs. S- LBP + RV septum: 17.6 ± 1.3 ms, *p* < 0.01). Anodal capture of the right bundle during selective LBP led to further improvements of biventricular activation and synchrony. BIVAT-90 were 48.2 ± 5.2 ms and significantly shorter than baseline and selective LBP with RV septum anodal capture, due to significantly shorter RVAT-95 (35.9 ± 5.4 ms, *p* < 0.01 vs. baseline). BIV synchrony was also better compared to RV septum anodal capture (BIVDI: 14.5 ± 1.6 ms, *p* < 0.01 vs. LBP + RV septum capture), and comparable to selective HBP (BIVDI: HBP 13.2 ± 1.3 ms, *p* = 0.13). Non-selective LBP rather than selective capture during RV anodal capture did not affect any of the metrics (*p* > 0.05).

## 4 Discussion

**FIGURE 9:**
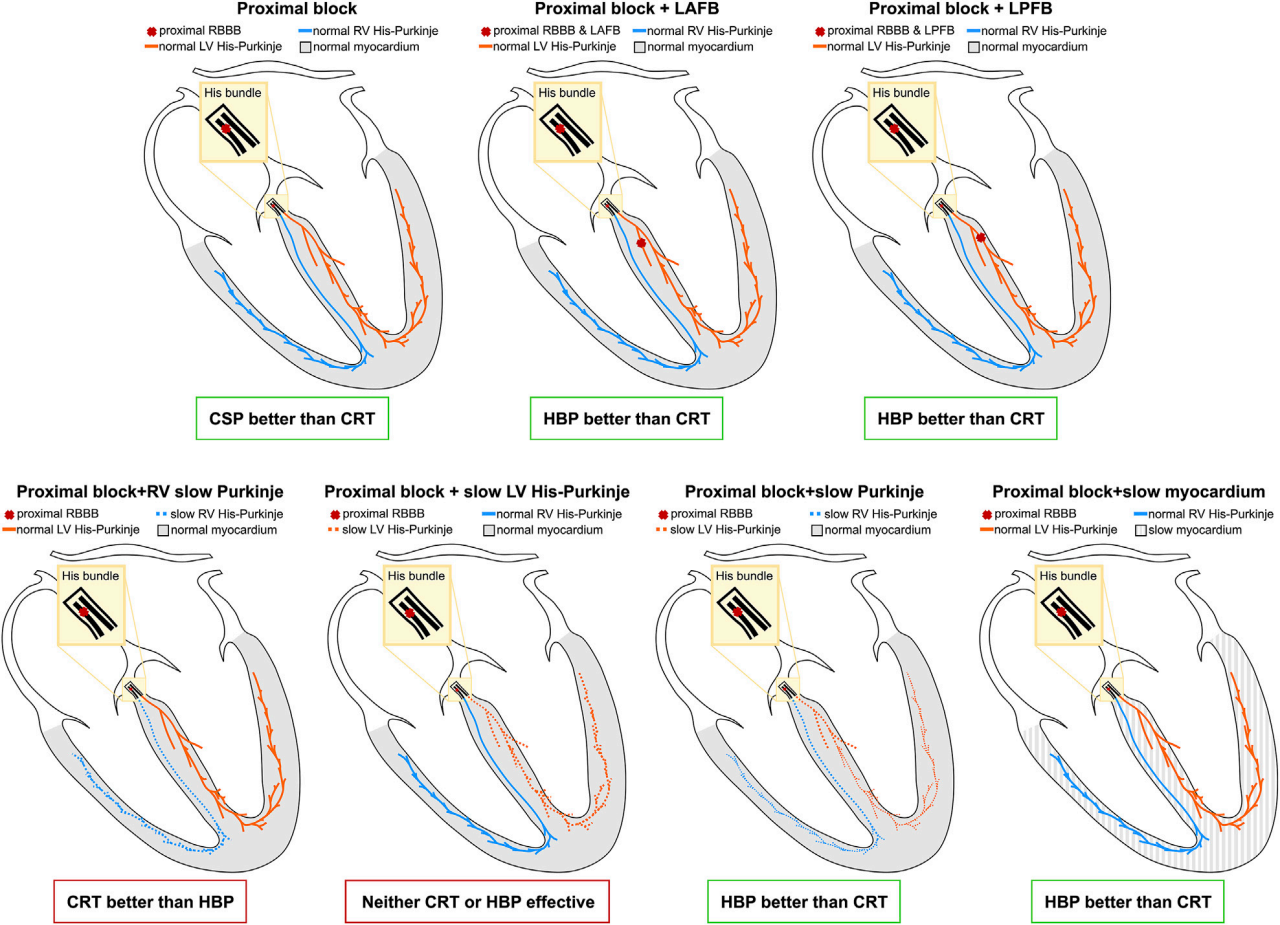
Results summary: The yellow area represents the His bundle, with a proximal block (red cross) introduced along the strands going to the right bundle. Additional blocks were introduced in the left anterior and posterior fascicles. The LV and the RV Purkinje system are represented in orange and the blue, and the ventricular myocardium is depicted in grey.

We performed an in-silico electrophysiology clinical trial to study the effect of standard CRT, HBP, and LBP on RBBB patients. We simulated the presence of RBBB combined with different conduction disturbances to see how they altered response to pacing. Figure 9 summarizes the results of our study. In patients with proximal RBBB and otherwise normal His-Purkinje system and myocardium, CRT and LBP were ineffective, while HBP improved activation. Similar observations applied when RBBB was combined with LAFB. On the other hand, patients with RBBB and LPFB responded to HBP, LBP and standard CRT, although HBP was still the most effective. Severe RV His-Purkinje conduction slowing combined with RBBB led to improved ventricular synchrony with CRT compared to HBP, while severe LV His-Purkinje conduction slowing made all pacing modalities ineffective due to severely prolonged LV activation. RBBB with slowed CV in the whole His-Purkinje system or the myocardium favored HBP over CRT, where LV and BIV activation were longer than baseline. We tested whether alternative ways of delivering LBP could improve RV activation and therefore improve overall ventricular synchrony. Non-selective LBP was comparable to selective LBP. On the other hand, selective LBP delivered in combination with anodal capture of the RV septum or the right bundle led to significantly improved ventricular activation compared to LBP alone.

Many large, multicenter clinical trials have proved that CRT is more effective in LBBB than RBBB patients. (Hara et al., 2012; Auricchio et al., 2014; Pastore et al., 2018; Henin et al., 2020). Despite this, a large proportion of RBBB patients are delivered with CRT to attempt to treat dyssychrony and improve cardiac function. In agreement with our study, Pastore et al. (2018) reported that patients with typical RBBB were less likely to respond to CRT compared to patients with atypical RBBB, where RBBB might mask some additional dyssynchrony. For instance, RBBB patients with LV dyssynchrony were shown to benefit from CRT, while in patients with RBBB and no or small LV dyssynchrony, CRT prolonged LV activation. (Salden O. A. E. et al., 2020). Our simulations show that only in presence of RBBB and LPFB or severely slow RV His-Purkinje conduction slowing, CRT improved BIV activation, while all other types of dyssynchrony did not favor CRT. Our results explain the variation in outcomes in patients with RBBB, and provide insight into which RBBB patient subgroups are likely to respond to CRT.

HBP is emerging as an alternative way of delivering CRT in patients where standard CRT is not feasible or ineffective. HBP safety and efficacy has been investigated and confirmed by small clinical trials on LBBB patients, (Lustgarten et al., 2015; Arnold et al., 2018), while there is a lack of data on the effect of HBP in the presence of RBBB. Nevertheless, if the site of block is proximal enough, HBP theoretically represents the most physiological method to deliver resynchronization in RBBB patients, potentially leading to perfect correction of RBBB morphology. Sharma et al. (2018) reported significant QRS narrowing in RBBB patients following successful delivery of HBP, consistent with our results. Despite these promising preliminary results, future efforts to investigate feasibility of HBP on RBBB patients are needed to extend HBP delivery to non-LBBB patient groups, with particular care in identifying dyssynchronies that are less likely to favor HBP efficacy.

LBP can be used as an alternative to HBP, when HBP cannot be delivered or requires high pacing thresholds to correct delayed LV activation in LBBB patients. Clinical and computational studies have shown that LBP can be as effective as HBP when AV delay optimization is possible, aiming to shorten RV activation times through the patient’s intrinsic activation travelling down the viable fibers of the right bundle. (Strocchi et al., 2020b; Lin et al., 2020). This is however not possible in RBBB patients. In agreement with our study, Vijayaraman et al. (2022) showed that LBP led to small changes in QRS duration (from 156 ± 20 ms to 150 ± 24 ms) in RBBB patients. On the other hand, HBP led to significant QRS narrowing. The authors however also reported that 33% of patients had complete RBBB correction following LBP, attributing this to two potential factors: non-selective LBP or anodal capture of the RV septum. Our simulations show that, while non-selective LBP would not explain improved RV activation compared to selective LBP, anodal capture of the RV septum during LBP could cause RBBB partial correction, with additional benefits induced by anodal capture of the right bundle. These results could be of particular interest with the advancement of leadless pacing. The Wise-CRT system (EBR Systems Inc., Sunnyvale CA) is a commercially available LV leadless pacing system that requires the co-implant of an RV lead delivering continuous pacing (Wijesuriya et al., 2022). Our results suggest that, by placing the RV lead in the RV septum potentially targeting the right bundle while delivering LBP through the leadless electrode, the Wise-CRT system could benefit RBBB patients. Although LBP alone might not constitute a viable method to deliver CRT in RBBB patients, its delivery could still be adapted and extended to this patient group.

### 4.1 Limitations

Although in-silico trials provide a systematic comparison between different CRT delivery methods, they rely on models with limitations. We assume that acute electrical response implies long-term benefits. Additional factors might affect response to therapy, but a review of CRT clinical trials showed that QRS narrowing was more significant in responders compared to non-responders (Bryant et al., 2013). Nevertheless, a future computational study considering mechanical as well as electrical synchrony induced by pacing would be of great interest, and might provide additional information about functional response to CRT. Furthermore, when simulating HBP, we assume perfect correction of RBBB through selective capture, while a proportion of patients are delivered with non-selective HBP, with clinical trials reporting selective capture achieved in between 6% and 100% of patients (Lewis et al., 2019). Therefore, our results might overestimate the electrical synchrony induced by HBP. We have performed simulations with non-selective HBP for all conduction disturbances, and provided a comparison with selective HBP in Section 3 of the Supplement. Non-selective pacing was equivalent to selective pacing for all patient groups but RBBB combined with LPFB and with severely slow His-Purkinje system. Nevertheless, early activation of the area around the His bundle might lead to changes in strains, therefore affecting mechanical synchrony, that this study does not account for.

Our models include synthetically generated His-Purkinje systems that did not replicate the conduction system of a specific patient. Nevertheless, we were able to simulate the main features of RBBB activation, as shown in the Supplement.

Finally, we simulated only ventricular activation times without propagation of the electrical activation in the torso, therefore not providing simulated surface ECGs. However, the torso geometries were not available for these patients. Therefore, simulating ECGs with sufficient accuracy would constitute a challenge that was outside the scope of this study.

Regardless of its limitations, our computational study provides a systematic comparison between different pacing modalities in the presence of RBBB combined with different conduction disturbances, showing which RBBB subgroups could respond to CRT, HBP, and LBP.

## 5 Conclusion

Patients with RBBB significantly benefit from HBP but not CRT or LBP, which preserves delayed RV activation. LBP delivered in combination with anodal capture of the RV septum shortens RV activation, leading to overall improved synchrony. When RBBB is concomitant with LPFB, CRT improves activation, but HBP is still more effective thanks to RBBB correction. Severely slow RV His-Purkinje CV with proximal RBBB favors CRT over HBP, while severely slow LV His-Purkinje CV makes both CRT and HBP ineffective. When conduction slowing affects the whole His-Purkinje system or the ventricular myocardium, HBP is more effective than CRT.

## Data Availability

The raw data supporting the conclusion of this article will be made available by the authors, without undue reservation.
