# Supplementary material for: Comparison between conduction system pacing and cardiac resynchronization therapy in right bundle branch block patients
Source: Front Physiol. 2022 Sep 21;13:1011566. doi: 10.3389/fphys.2022.1011566 (PMC9532840; doi:10.3389/fphys.2022.1011566)
Supplement: Supplementary file 1 [file DataSheet1.pdf]

Supplementary Material

1 His-Purkinje Network Generation

The His-Purkinje network generation method was based on (Gillette et al., 2021, 2022) and is described in detail in the original publication. Figure 1 shows the inputs for the His-Purkinje network generation pipeline (top row). The Purkinje tree is grown on the left ventricle (LV) and on the right ventricle (RV) endocardial surfaces (red surfaces in Figure 1, top row), excluding the base, as this was previously shown to lead to more physiological sinus rhythm activation. (Strocchi et al., 2020) Universal ventricular coordinates (UVCs) were defined on the ventricles (Figure 1, top row), consisting of an apico-basal coordinate, ranging continuously from 0 to 1 from apex to base, a transmural coordinate, defined as 0 at the endocardium and 1 at the epicardium, and a rotational coordinate, rotating around the ventricles from -π to 0 from the LV free wall to the septum and then back from 0 to +π from the septum back to the LV free wall.

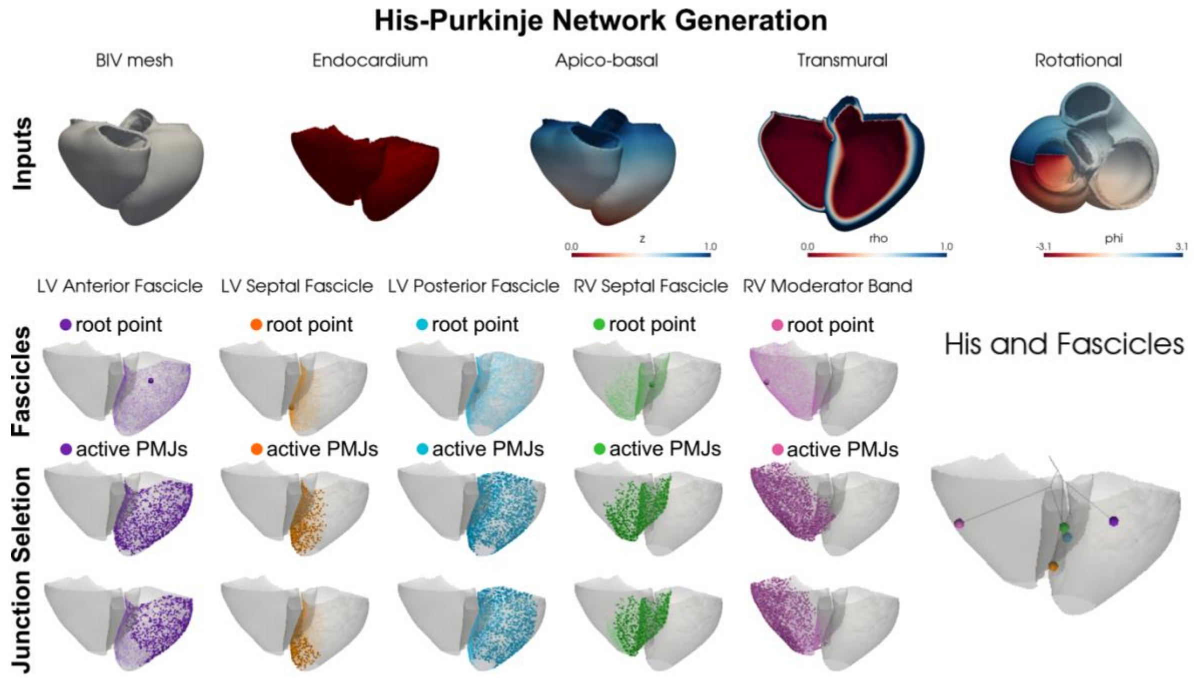

Figure 1 His-Purkinje network generation pipeline. The first row shows the inputs for the His-Purkinje Network generation (from left to right): a biventricular mesh, the left and right ventricular endocardial surfaces the network is grown on, an apico-basal universal ventricular coordinate (UVC), ranging between 0 at the apex and 1 at the base, a transmural UVC, varying from 0 to 1 from the endocardium to the epicardium and a rotational UVC from -π to +π around the ventricles. The second row shows the root points for the five fascicles included in the modes. The third and rows represent all Purkinje-myocardium junctions (PMJs) and the final PMJs, remaining after deactivating the redundant PMJs. On the right, a picture of the His and fascicles, showing the root points as colored spheres.

The model accounts for three LV fascicles (colors refer to Figure 1, second to fourth row): anterior (purple), septal (orange) and posterior (light-blue), and for two RV fascicles: septal (green) and moderator band (pink). The location of the root points (second row, Figure 1) was provided in terms of UVCs and was based on early

activated areas in the Durrer maps. (Durrer et al., 1970) The root points were then used to grow five independent networks that were joined to the His as shown in Figure 1, bottom-right. The His bundle is formed by filaments bundled together and insulated within a common cable.(Vijayaraman et al., 2018) These filaments are predestined to either the left bundle or the right bundle. To represent this anatomical property of the His bundle, we duplicated the His bundle segments in our Purkinje networks. One strand continues into the left bundle and the other continues into the right bundle.

The trees associated with the fascicles are grown independently from each other and can therefore overlap. This would not make a difference in sinus rhythm simulations, where the stimulus spreads from the His, fascicles, Purkinje and finally to the myocardium through the Purkinje-myocardium junctions (PMJs), e.g. the terminal points of the His-Purkinje network, as each node can activate only once. On the other hand, during pacing, additional PMJs can affect the simulation as the stimulus can enter the wrong fascicle. To prevent this, we deactivated redundant PMJs. First, the PMJs associated with each fascicle were found (Figure 1, third row). We then identified the portions of LV and RV endocardial surfaces that were covered by each fascicle tree, and we identified areas where the trees overlapped. An Eikonal simulation was run on the tree associated with each fascicle by stimulating the root point to find when each terminal point would activate. In areas where the fascicle trees overlapped, only the PMJs with the shortest activation time were connected with the surrounding myocardium and were therefore activated. The PMJs remaining active after this procedure are shown in Figure 1, bottom row. The image shows that there is no overlap between active PMJs belonging to different fascicles.

The timing of the first activations from the fascicles to ventricular myocardium was based on the Durrer maps. (Durrer et al., 1970) The first LV activation occurs at the three simultaneous fascicle locations (anterior, septal and posterior). Therefore, we computed the conduction velocity (CV) of each LV fascicle separately to guarantee simultaneous activation of the root points of the fascicles. According to the Durrer maps, the RV fascicles activate about 10 ms later than the LV fascicles. Therefore, the CV of the RV fascicles was computed to achieve activation of the RV fascicles root points 10 ms later than the LV.

This network generation pipeline was applied to all twenty-four patient-specific meshes. Proximal RBBB was simulated by disconnecting the left bundle branch from the RV Purkinje network along the His.

2 Baseline Simulated Activation

To show that our model replicates RBBB activation patterns, we show simulated activation times on all patient hearts during RBBB and compare them with available reported features in the literature. Figure 2 and 3 show activation times simulated on all twenty-four patient hearts at RBBB baseline in an anterior and a posterior view, respectively. Red and blue areas represent early and late activated regions, respectively. For all patients, the LV is activated early thanks to the intact LV His-Purkinje. The activation wave then slowly travels to the RV, in agreement with RBBB activation pattern reported in RBBB patients (Figure 1 in (Fantoni et al., 2005) and Figure 1A in (Strik et al., 2018)). (Strik et al., 2018) also reported a total activation time of 111±27 ms in four RBBB patients derived from electrocardiographic imaging. To measure LV-RV synchrony, the authors computed ventricular electrical uncoupling (VEU) as the difference between mean LV epicardial activation times and mean RV epicardial activation time. Therefore, positive values indicate RV pre-excitation and delayed LV activation, while negative values indicate LV pre-excitation and delayed RV activation. In this study, RBBB patients had a VEU of -36±12 ms, in keeping with delayed RV activation. Our simulations result in a total ventricular activation time of 107±10 ms and a VEU of -23±4 ms, consistent with the data reported in (Strik et al., 2018), although simulated mean VEU was slightly bigger than reported average VEU. This might be due to our cohort having larger LV size compared to the cohort in (Strik et al., 2018), which was based only on four RBBB patients.

We also provide simulated activation times for proximal RBBB concomitant with LAFB or LPFB for all patients in Figure 4, 5, 6 and 7. As expected, RBBB combined with LAFB and LPFB leads to delayed LV activation in the anterior or the posterior regions of the LV free wall, in addition to delayed RV activation. This is consistent with activation maps in Figure 5 in (Fantoni et al., 2005), where the authors show the activation pattern in one patient with RBBB masking left bundle branch block. In this patient, the LV was as delayed as the RV, consistently with the activation maps simulated for RBBB concomitant with LAFB (Figure 4 and 5) and LPFB (Figure 6 and 7).

These observations show that our models are able to simulate RBBB activation patterns and activation metrics reported in the literature, and to replicate activation time patterns when RBBB is concomitant with additional LV conduction disturbances.

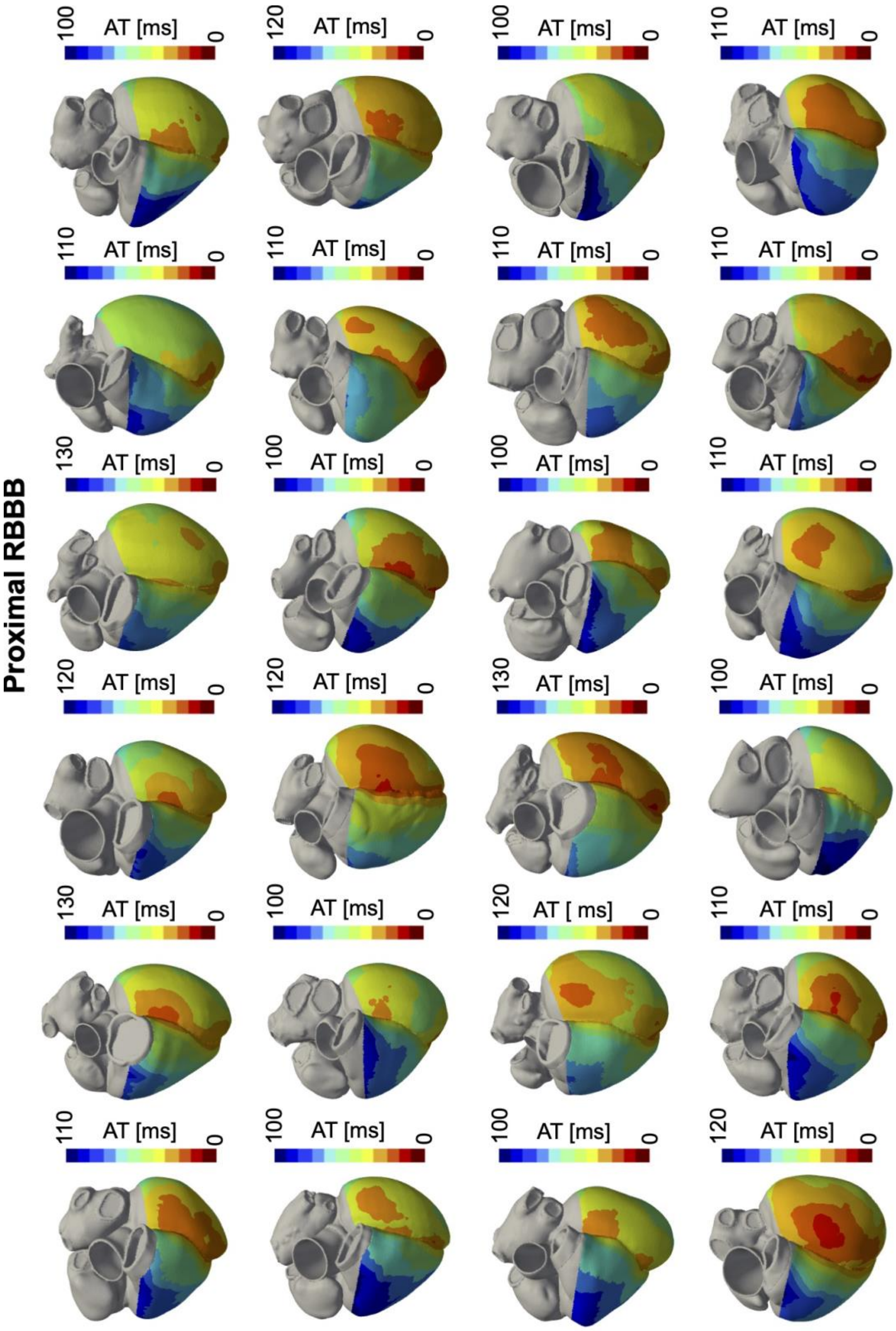

Figure 2 Distribution of simulated activation times (AT) during proximal RBBB showed on an anterior view of all twenty-four patient geometries.

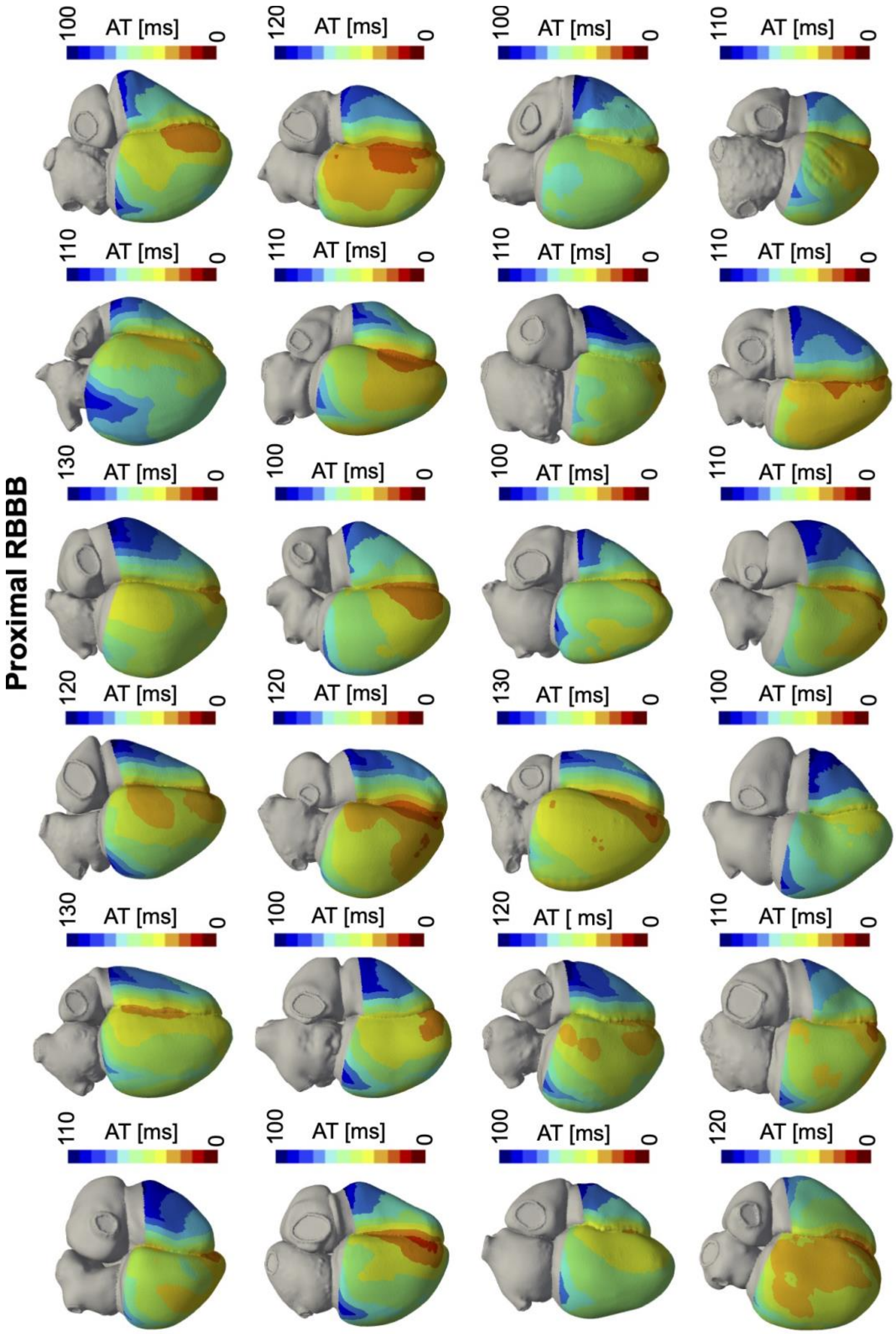

Figure 3 Distribution of simulated activation times (AT) during proximal RBBB showed on a posterior view of all twenty-four patient geometries.

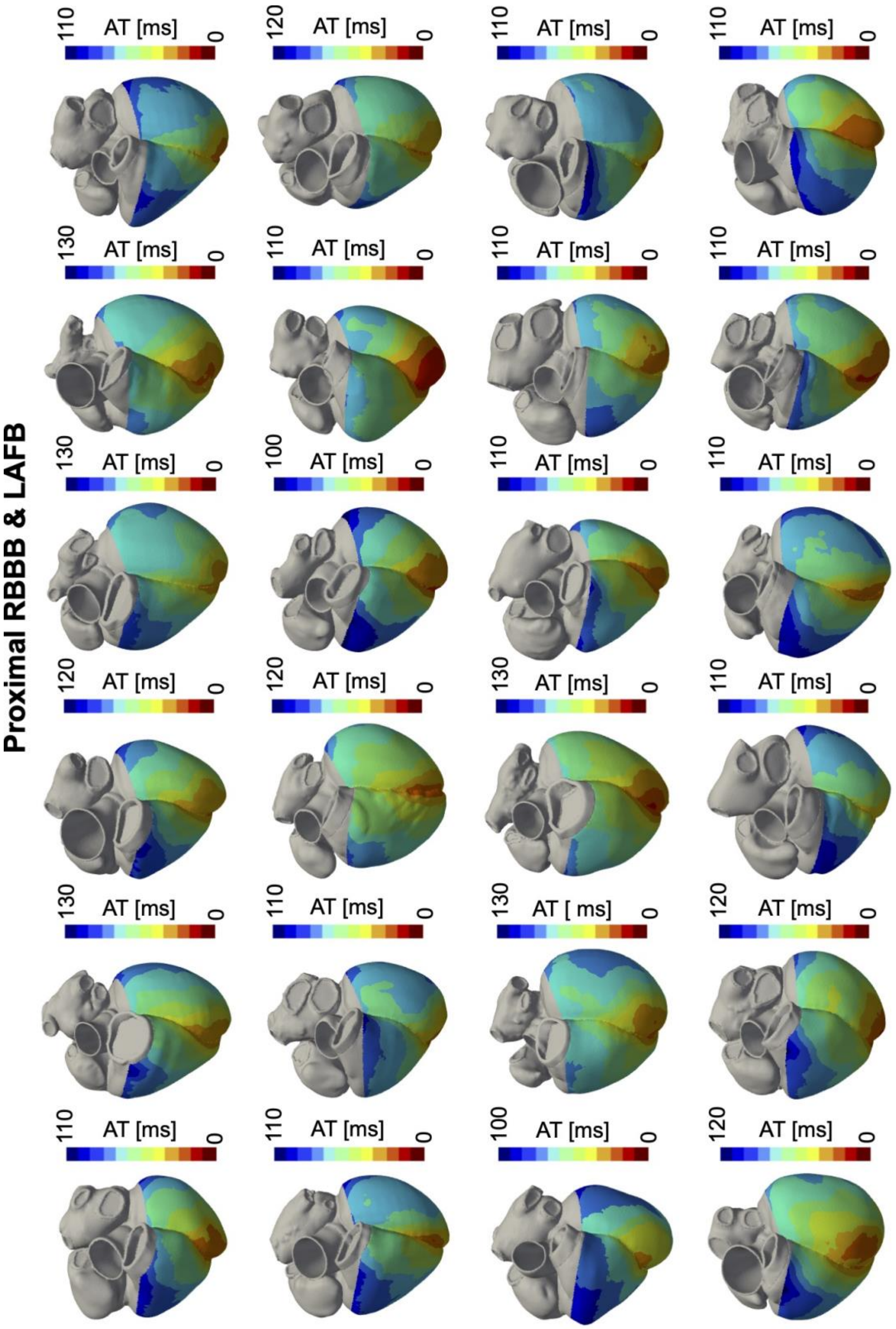

Figure 4 Distribution of simulated activation times (AT) during proximal RBBB and LAFB showed on an anterior view of all twenty-four patient geometries.

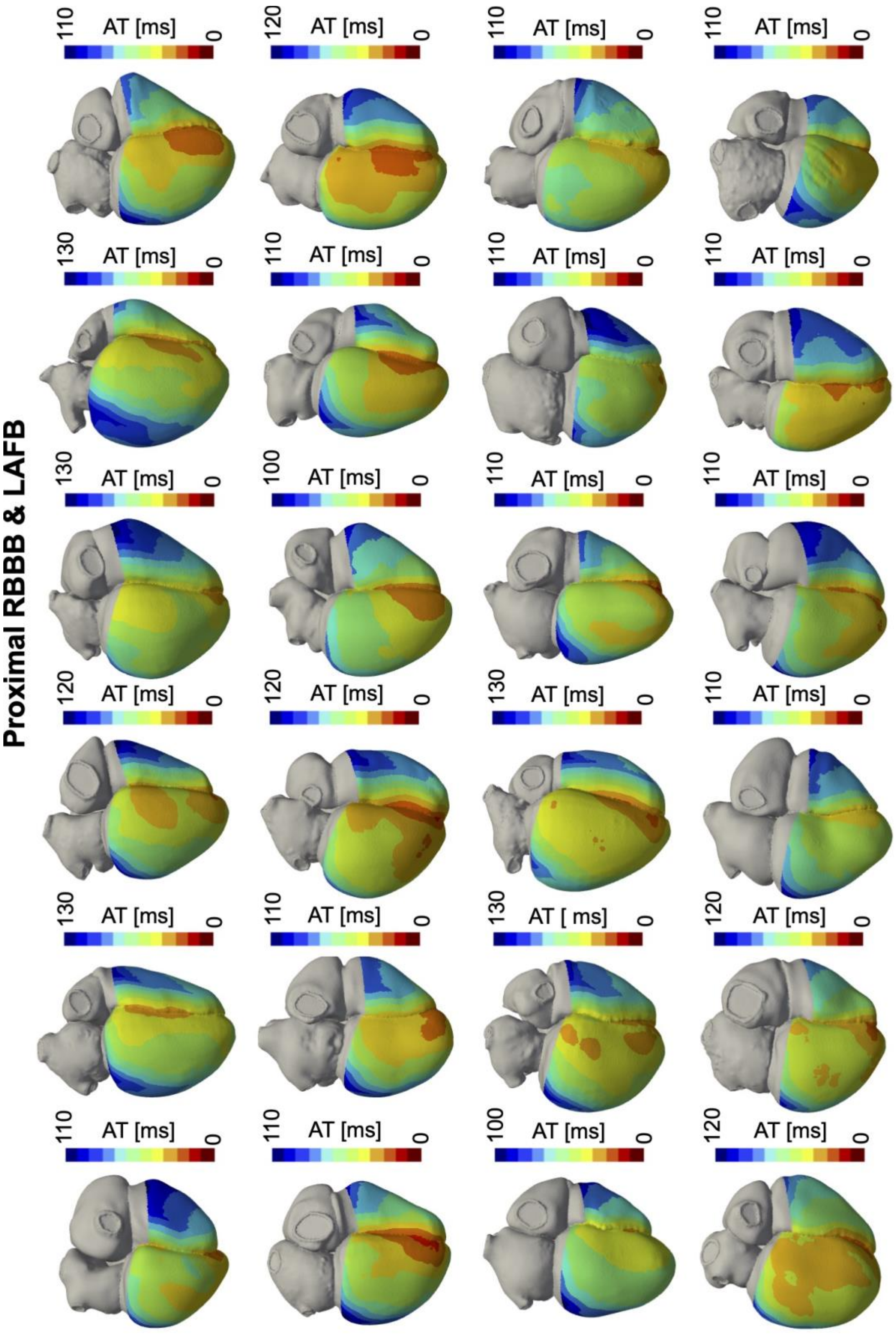

Figure 5 Distribution of simulated activation times (AT) during proximal RBBB and LAFB showed on a posterior view of all twenty-four patient geometries.

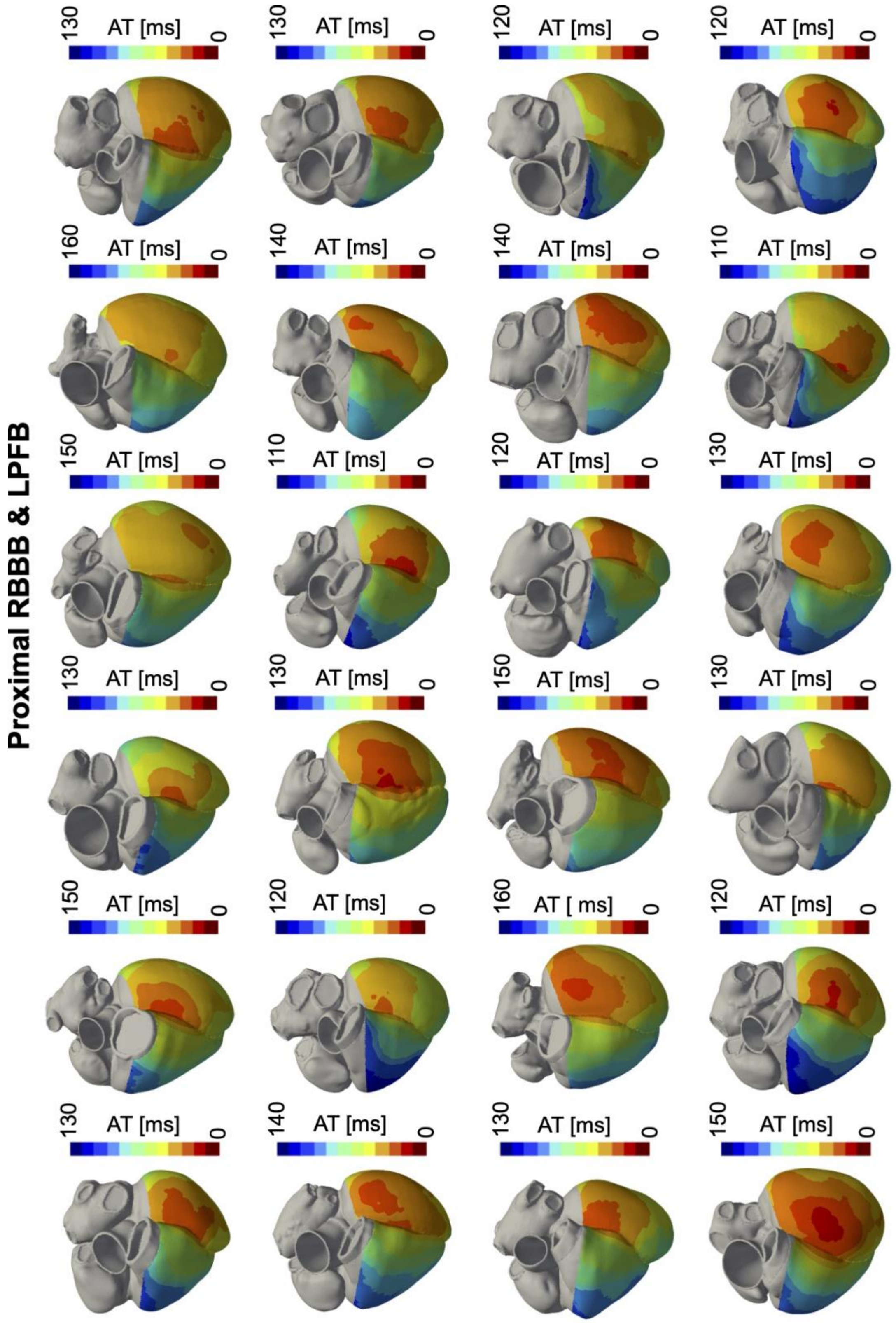

Figure 6 Distribution of simulated activation times (AT) during proximal RBBB and LPFB showed on an anterior view of all twenty-four patient geometries.

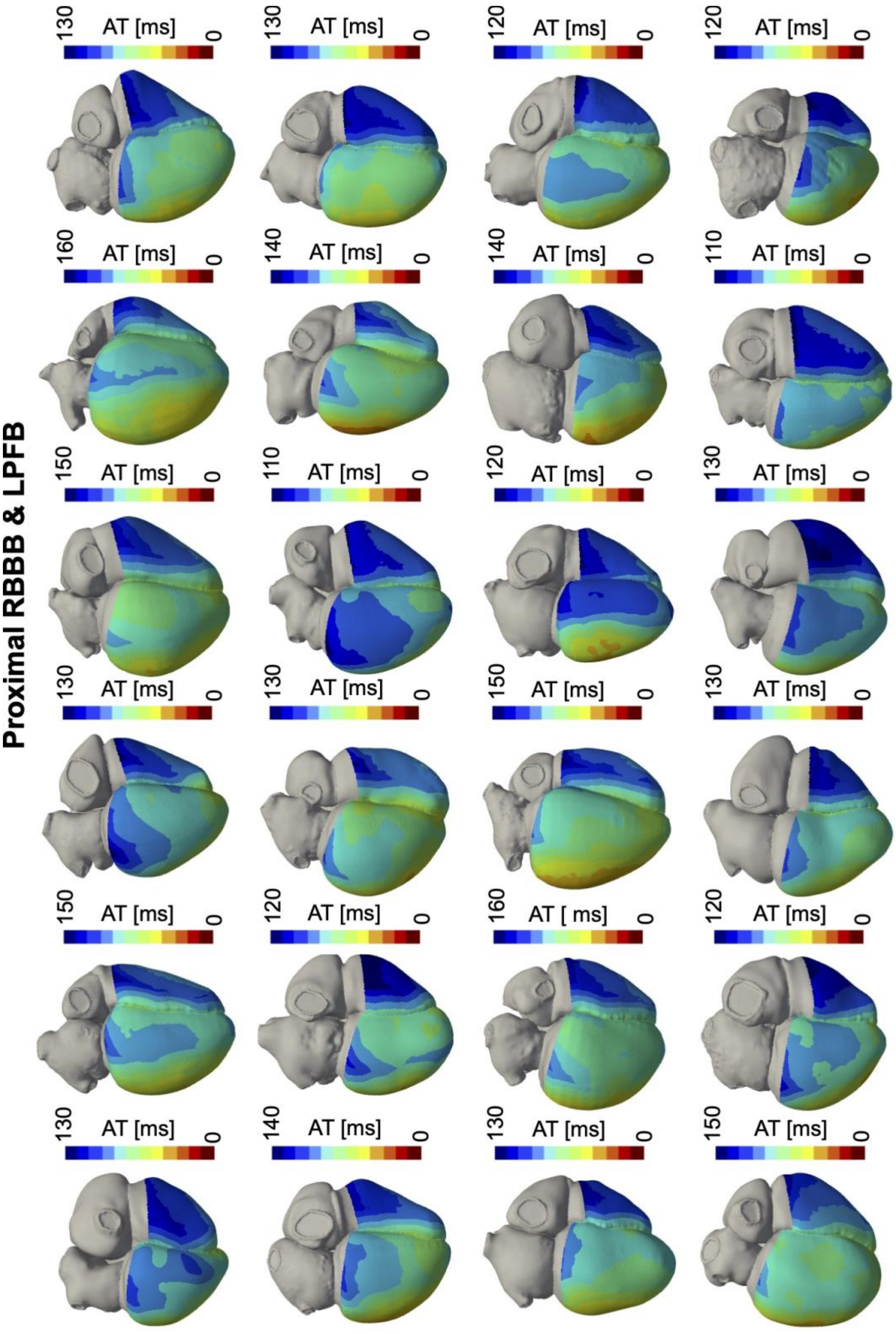

Figure 7 Distribution of simulated activation times (AT) during proximal RBBB and LPFB showed on a posterior view of all twenty-four patient geometries.

3 Selective vs non-selective His bundle pacing

His bundle pacing (HBP) is, in principle, the best way to deliver resynchronization in patients with proximal block as it has the potential to completely reverse the block and restore the native synchronous activation of the patient. However, HBP is difficult to perform, and a proportion of patients are delivered with non-selective HBP rather than selective HBP.

To study the effect of non-selective vs selective His bundle capture on our results, we performed simulations with non-selective HBP by extending the HBP stimulus to the surrounding myocardium. Then, we compared LVAT-95, LVDI, RVAT-95, RVDI, BIVAT-90 and BIVDI between baseline, CRT, selective HBP, non-selective HBP and selective LBP for all conduction disturbances considered in the manuscript (Figure 8 and 9).

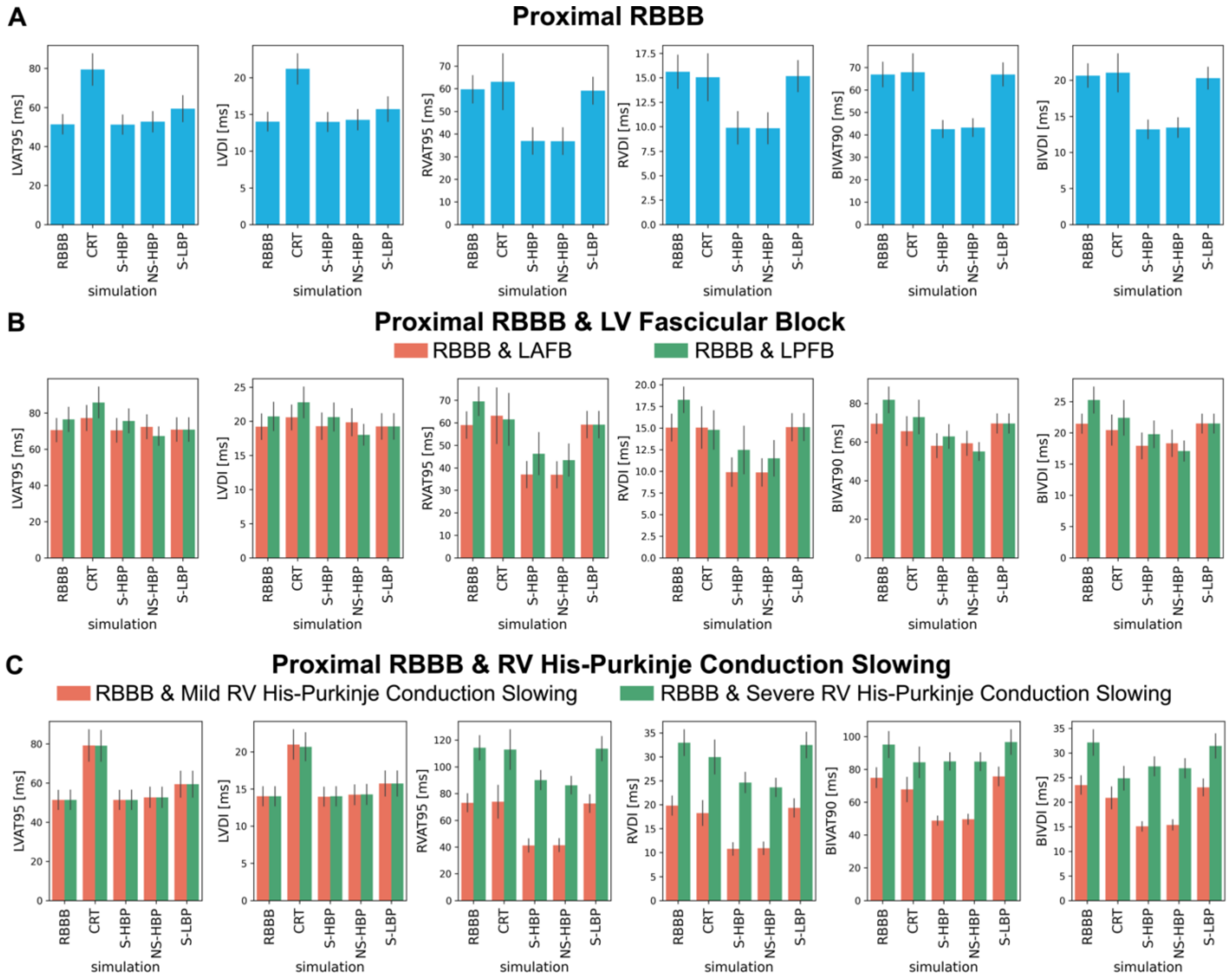

Figure 8 Response with RBBB, RBBB+LAFB, RBBB+LPFB and RBBB+RV His-Purkinje conduction slowing: LVAT-95, LVDI, RVAT-95, RVDI, BIVAT-90 and BIVDI simulated in the presence of proximal RBBB combined with otherwise normal His-Purkinje system (**A**), left anterior or posterior fascicular block (**B**) and mild or severe RV His-Purkinje conduction slowing

(**C**).Activation was simulated during baseline, standard CRT, selective HBP (S-HBP), non-selective HBP (NS-HBP) and selective LBP (S-LBP). Results are presented as mean±standard deviation.

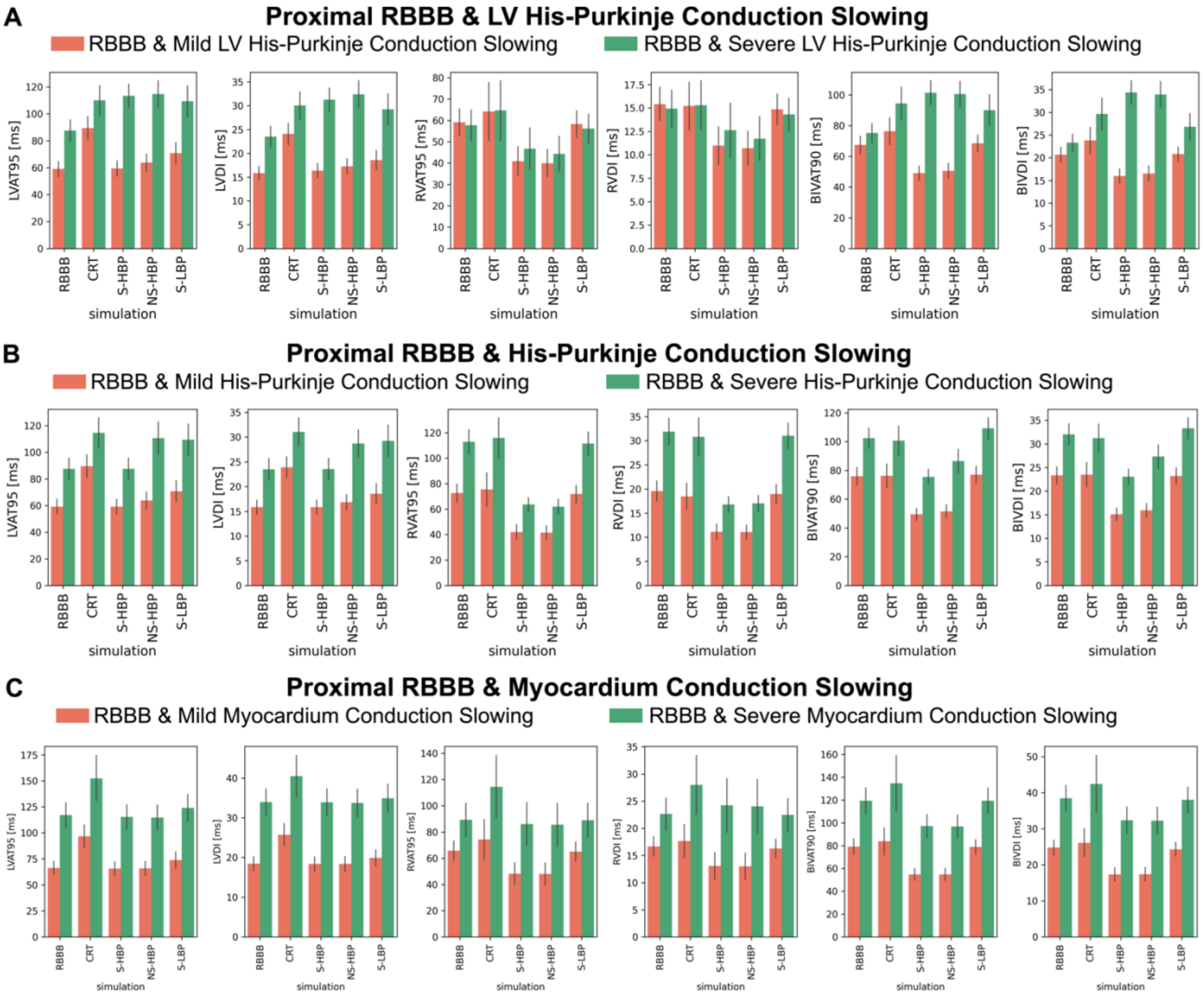

Figure 9 Response with RBBB+LV His-Purkinje, LV and RV His-Purkinje or myocardium conduction slowing: LVAT-95, LVDI, RVAT-95, RVDI, BIVAT-90 and BIVDI simulated in the presence of proximal RBBB combined with mild or severe LV His-Purkinje conduction slowing (**A**), mild or severe whole His-Purkinje conduction slowing (**B**) and mild or severe myocardium conduction slowing (**C**).Activation was simulated during baseline, standard CRT, selective HBP (S-HBP), non-selective HBP (NS-HBP) and selective LBP (S-LBP). Results are presented as mean±standard deviation.

During proximal RBBB with otherwise normal His-Purkinje system (Figure 8A), there was no significant difference between selective and non-selective HBP for all metrics. Therefore, all considerations done in the manuscript about this patient group is not affected by the type of His bundle capture. Similarly, when RBBB is concomitant with LAFB, non-selective HBP was equivalent to selective HBP in terms of LV, RV or BIV activation times (Figure 8B). On the other hand, RBBB combined with LPFB benefits from non-selective capture of the His bundle. This is because selective HBP is unable to correct the LPFB because the pacing stimulus is placed upstream from the block, while non-selective HBP activates the posterior wall of the septum early, improving

LV (LVAT-95: 85.8±8.5 ms vs 75.6±6.6 ms, P<0.01) and BIV activation times (BIVAT-90: 72.9±8.6 ms vs 62.9±6.2 ms, P<0.01). When RBBB is concomitant with mild or severely slow RV His-Purkinje system (Figure 8C), non-selective capture did not change response compared to selective HBP.

When RBBB is concomitant with mild or severe LV His-Purkinje conduction slowing (Figure 9A), LV, RV and BIV activation times are largely unaltered between selective or non-selective HBP, with all differences being non-statistically significant for all activation metrics. When the whole His-Purkinje system is affected by mild slowing (Figure 9B, orange), non-selective HBP is again equivalent to selective HBP in terms of all metrics. On the other hand, when the His-Purkinje conduction slowing is severe (Figure 9B, green), non-selective HBP is significantly worse than selective HBP in terms of LV (LVAT-95: 87.6±7.9 ms vs 110.5±12.2 ms, P<0.01) and BIV activation times (BIVAT-90: 75.5±5.2 ms vs 86.4±8.2 ms, P<0.01). This is because the stimulus activates a significant portion of the LV before it can travel across the His-Purkinje system and activate the rest of the LV. This leads to prolonged activation during non-selective HBP because a significant portion of the activation occurs through slow myocardium rather than the Purkinje. Finally, during RBBB concomitant with mild or severe myocardium conduction slowing (Figure 9C), non-selective HBP and selective HBP were equivalent in terms of all metrics.

For most of the RBBB patient groups we studied, selective and non-selective HBP were equivalent in terms of LV, RV and BIV activation. This is the case for proximal RBBB alone, or combined with LAFB, mild and severely slow RV His-Purkinje, LV His-Purkinje or myocardium conduction slowing. However, when RBBB was concomitant with LPFB, the non-selective HBP stimulus activates the posterior area of the septum, leading to improved activation compared to selective HBP. On the other hand, when the whole His-Purkinje system is severely slow, LV and BIV activation times are significantly prolonged with non-selective vs selective HBP. The effect of non-selective His bundle capture therefore changes depending on the RBBB patient subgroup.

4 References

Durrer, D., van Dam, R.T., Freud, G.E., Janse, M.J., Meijler, F.L., Arzbaecher, R.C., 1970. Total excitation of the isolated human heart. Circulation 41. https://doi.org/10.1161/01.CIR.41.6.899

Fantoni, C., Kawabata, M., Massaro, R., Regoli, F., Raffa, S., Arora, V., Salerno-Uriarte, J.A., Klein, H.U., Auricchio, A., 2005. Right and left ventricular activation sequence in patients with heart failure and right bundle branch block: A detailed analysis using three-dimensional non-fluoroscopic electroanatomic mapping system. J Cardiovasc Electrophysiol 16. https://doi.org/10.1046/j.1540-8167.2005.40777.x

Gillette, K., Gsell, M.A.F., Bouyssier, J., Prassl, A.J., Neic, A., Vigmond, E.J., Plank, G., 2021. Automated Framework for the Inclusion of a His–Purkinje System in Cardiac Digital Twins of Ventricular Electrophysiology. Ann Biomed Eng 49. https://doi.org/10.1007/s10439-021-02825-9

Gillette, K.K., Gsell, M.A.F., Neic, A., Manninger, M., Scherr, D., Roney, C.H., Strocchi, M., Prassl, A.J., Vigmond, E.J., Plank, G., 2022. A Personalized Real-time Virtual Model of Whole Heart Electrophysiology. Frontiers in Physiology (Accepted for publication).

Strik, M., Ploux, S., Huntjens, P.R., Nguyên, U.C., Frontera, A., Eschalier, R., Dubois, R., Ritter, P., Klotz, N., Vernooy, K., Haïssaguerre, M., Crijns, H.J.G.M., Prinzen, F.W., Bordachar, P., 2018. Response to cardiac resynchronization therapy is determined by intrinsic electrical substrate rather than by its modification. Int J Cardiol 270. https://doi.org/10.1016/j.ijcard.2018.06.005

Strocchi, M., Lee, A.W.C., Neic, A., Bouyssier, J., Gillette, K., Plank, G., Elliott, M.K., Gould, J., Behar, J.M., Sidhu, B., Mehta, V., Bishop, M.J., Vigmond, E.J., Rinaldi, C.A., Niederer, S.A., 2020. His-bundle and left bundle pacing with optimized atrioventricular delay achieve superior electrical synchrony over endocardial and epicardial pacing in left bundle branch block patients. Heart Rhythm 17. https://doi.org/10.1016/j.hrthm.2020.06.028

Vijayaraman, P., Chung, M.K., Dandamudi, G., Upadhyay, G.A., Krishnan, K., Crossley, G., Bova Campbell, K., Lee, B.K., Refaat, M.M., Saksena, S., Fisher, J.D., Lakkireddy, D., 2018. His Bundle Pacing. J Am Coll Cardiol. https://doi.org/10.1016/j.jacc.2018.06.017
